# Supplementary figures and images for: Plasmodium falciparum Guanylyl Cyclase-Alpha and the Activity of Its Appended P4-ATPase Domain Are Essential for cGMP Synthesis and Blood-Stage Egress
Source: mBio. 2021 Jan 26;12(1):e02694-20. doi: 10.1128/mBio.02694-20 (PMC7858053; doi:10.1128/mBio.02694-20)

# Supplementary Figure 1

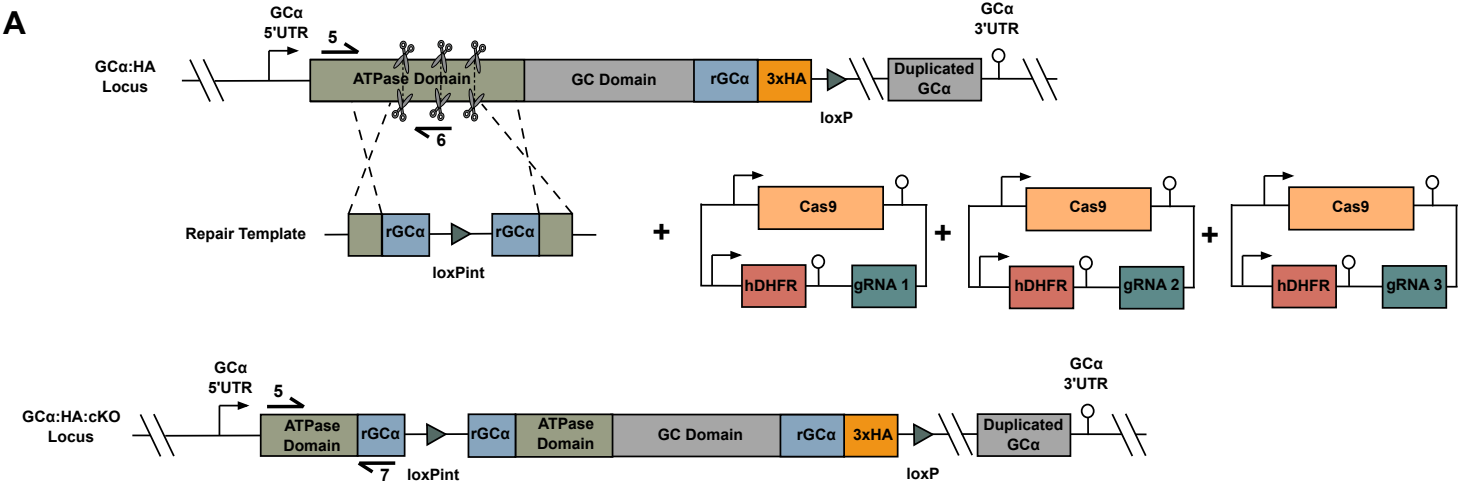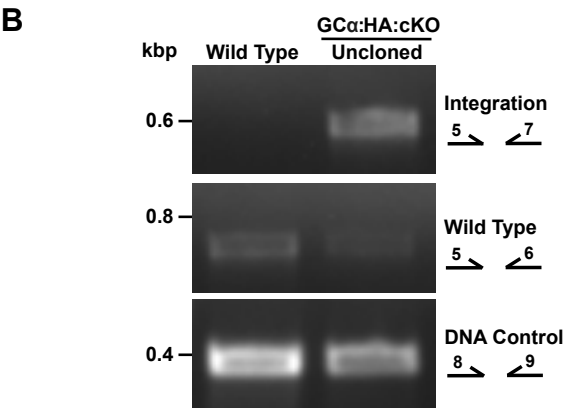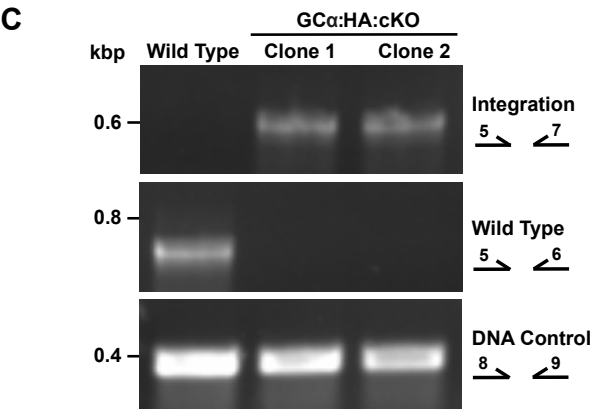

Supplement: FIG S1 [file mBio.02694-20-sf001.pdf]

Supplementary Figure 2

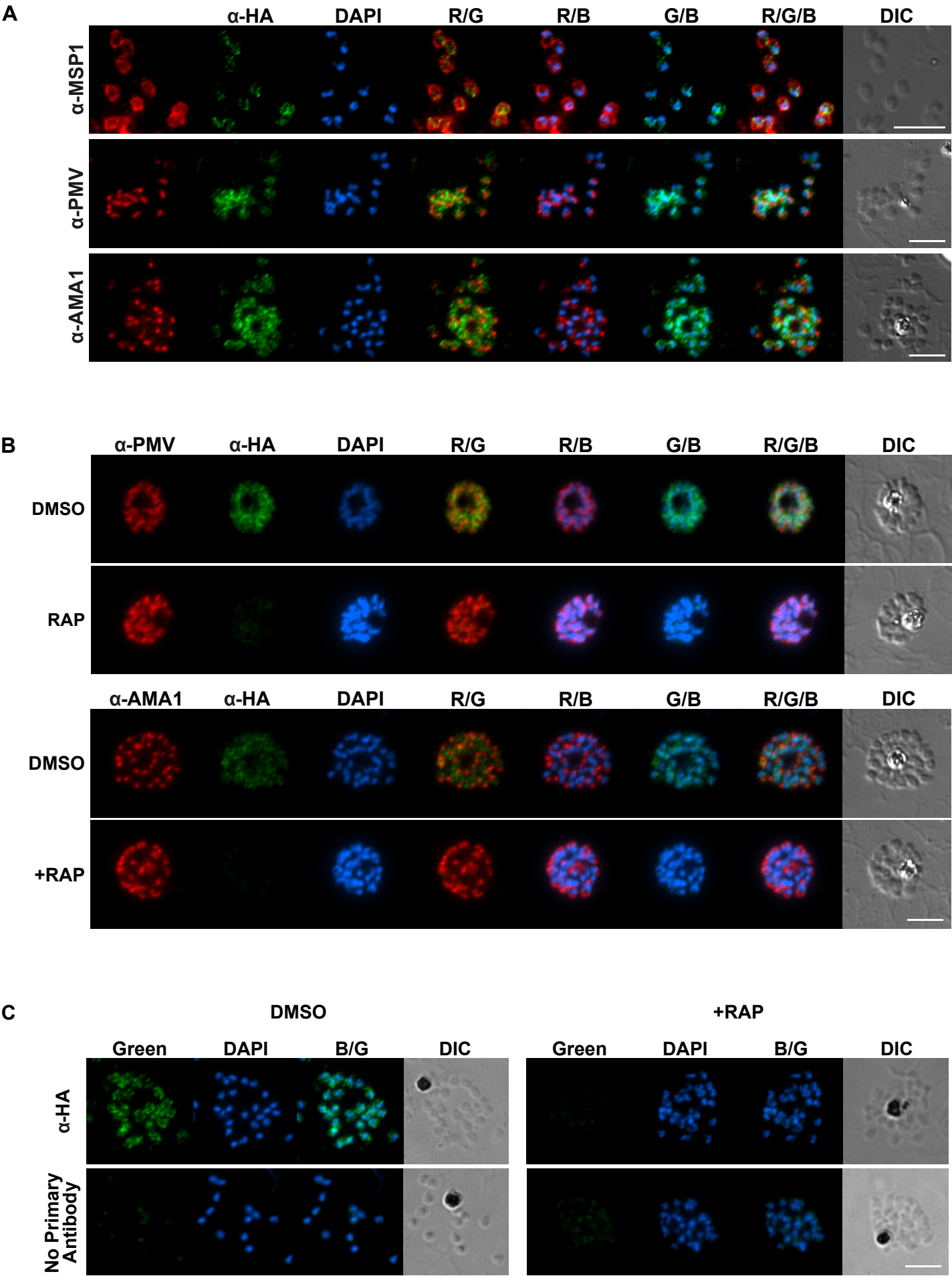

Supplement: FIG S2 [file mBio.02694-20-sf002.pdf]

# Supplementary Figure 3

A

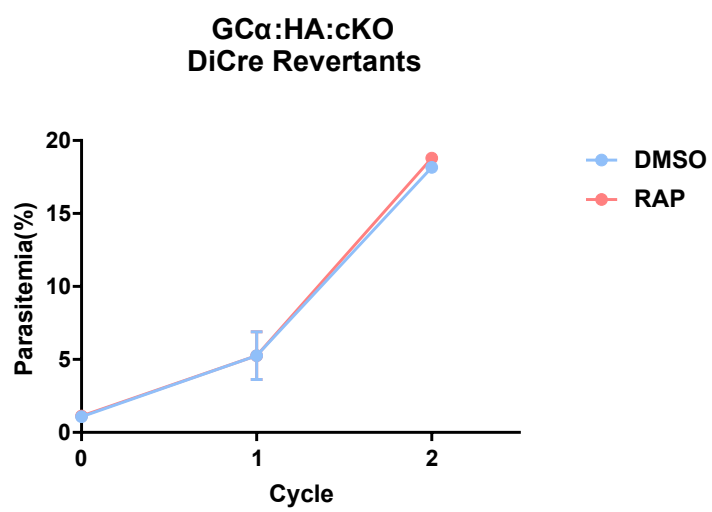

B

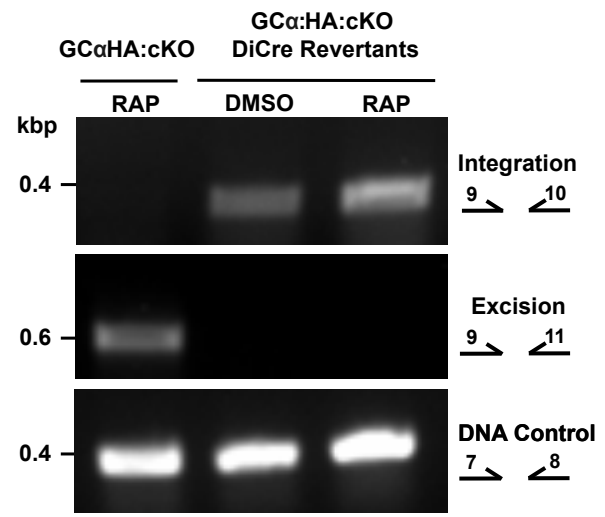

Supplement: FIG S3 [file mBio.02694-20-sf003.pdf]

# Supplementary Figure 4

**A**

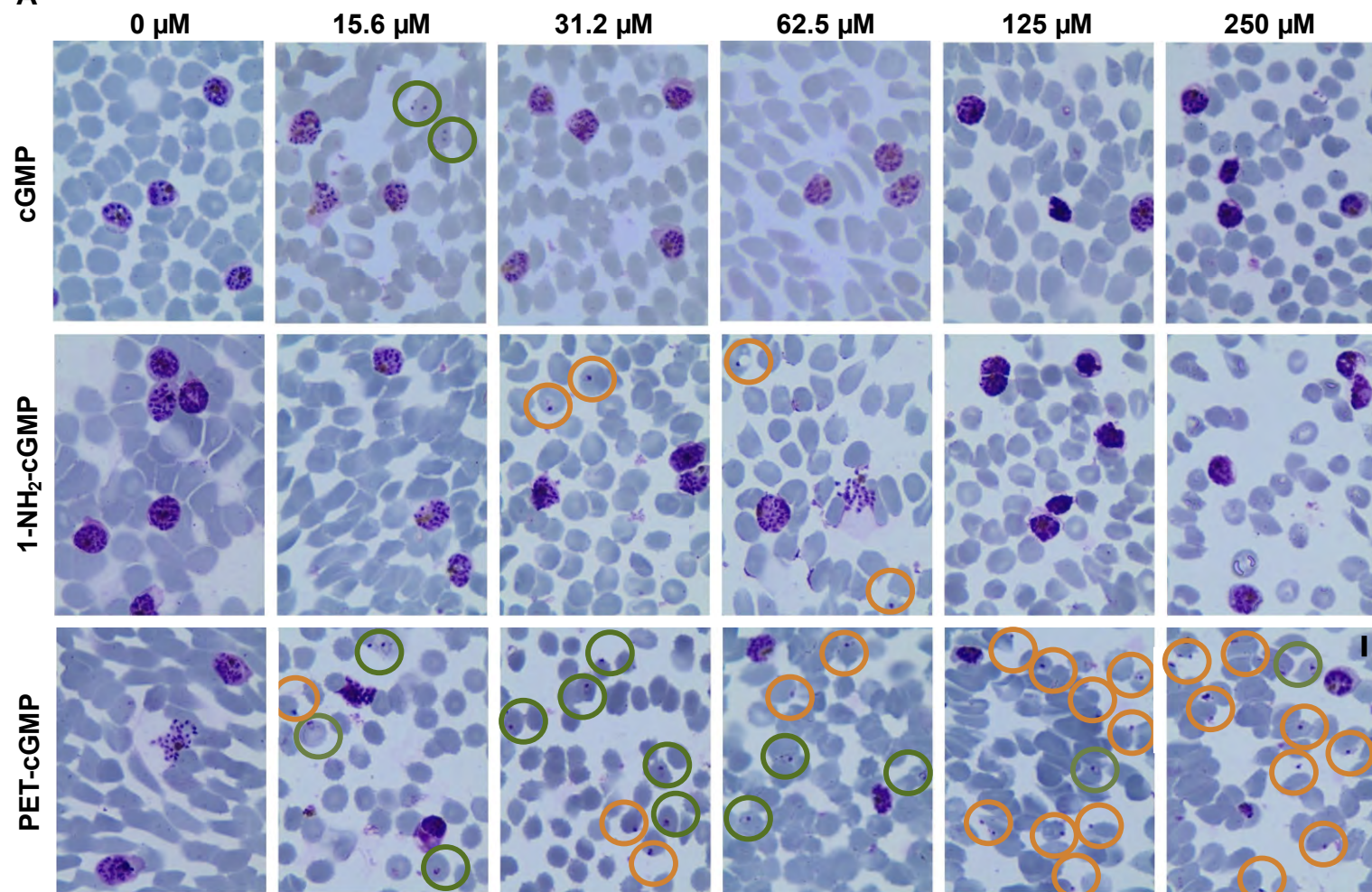

**B**

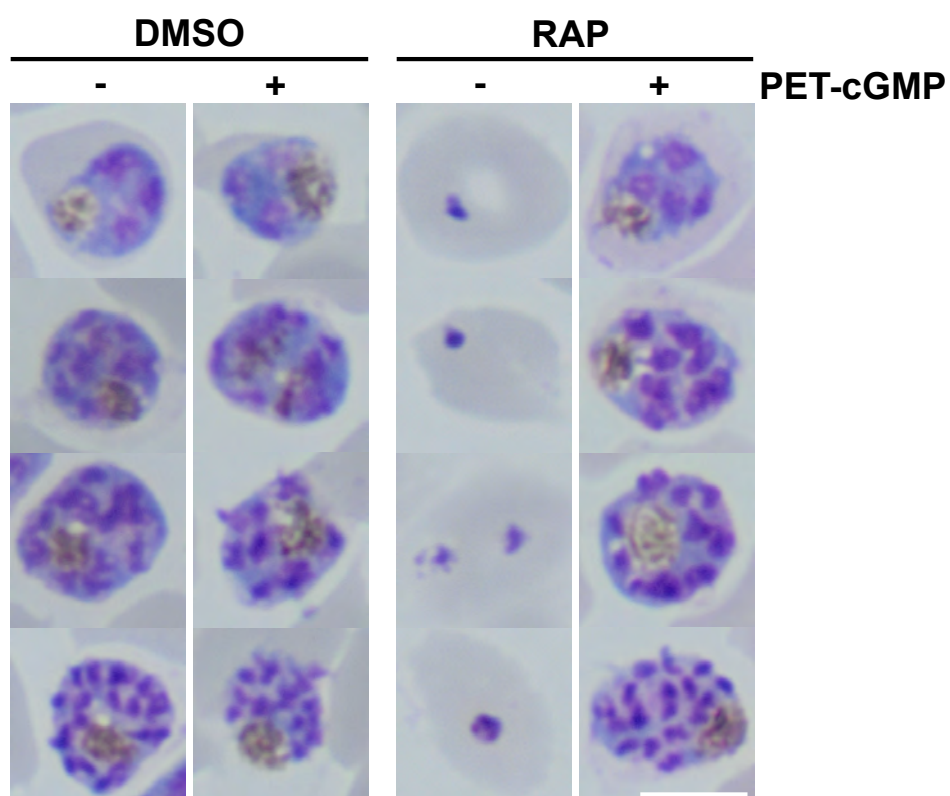

Supplement: FIG S4 [file mBio.02694-20-sf004.pdf]
