# Supplementary material for: Plasmodium falciparum Guanylyl Cyclase-Alpha and the Activity of Its Appended P4-ATPase Domain Are Essential for cGMP Synthesis and Blood-Stage Egress
Source: mBio. 2021 Jan 26;12(1):e02694-20. doi: 10.1128/mBio.02694-20 (PMC7858053; doi:10.1128/mBio.02694-20)

Supplementary Figure 5

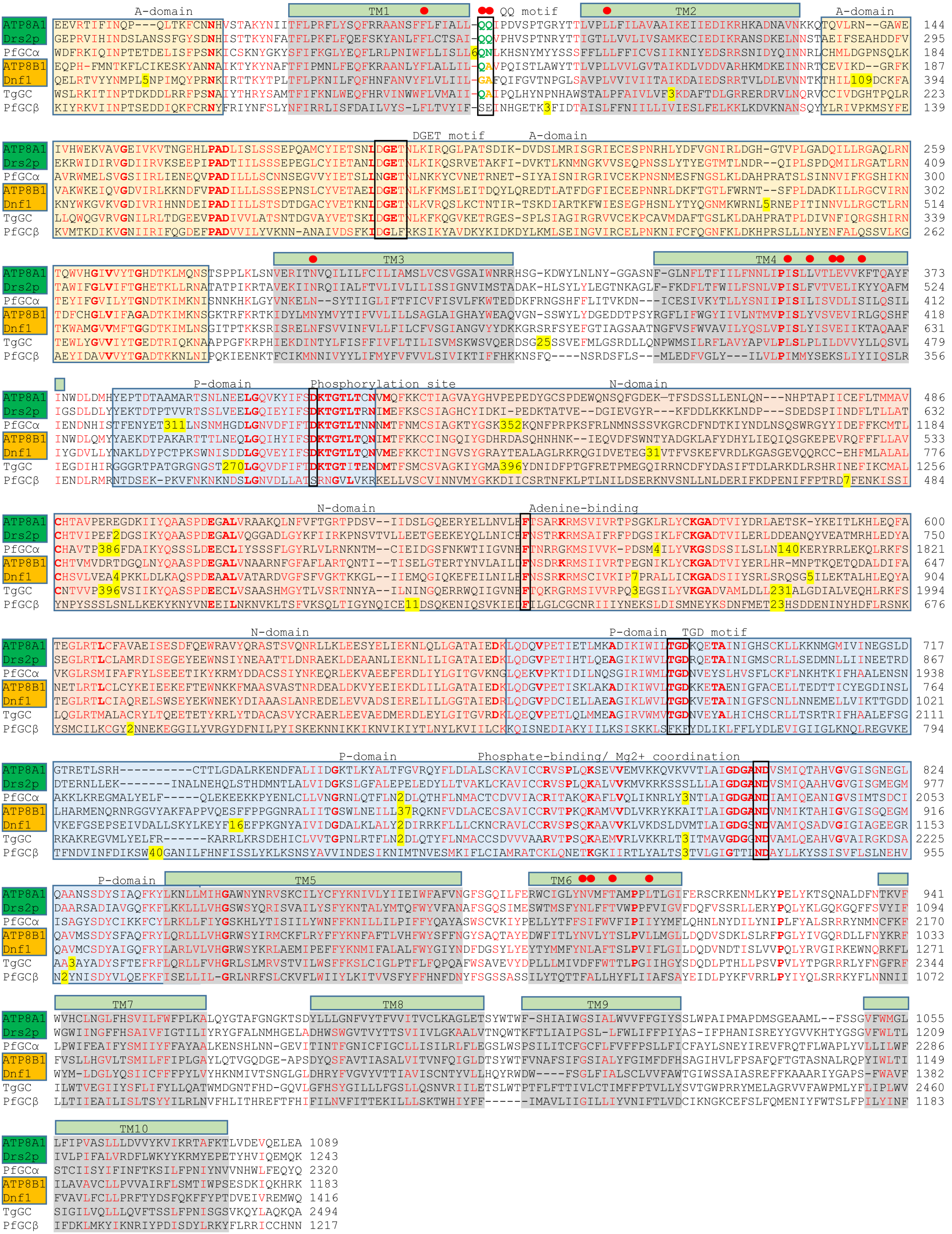

Supplement: FIG S5 [file mBio.02694-20-sf005.pdf]
