# Supplementary material for: Plasmodium falciparum Guanylyl Cyclase-Alpha and the Activity of Its Appended P4-ATPase Domain Are Essential for cGMP Synthesis and Blood-Stage Egress
Source: mBio. 2021 Jan 26;12(1):e02694-20. doi: 10.1128/mBio.02694-20 (PMC7858053; doi:10.1128/mBio.02694-20)
Supplement: TABLE S1 [file mBio.02694-20-st001.pdf]

Supplementary Table 1

Primer Number	Type	Primer Sequence 5' to 3'
1	F	ATTGAACATAATGAAGAAGATTACA
2	R	ATAACTTCGTATAATGTATGCTATAC
3	F	CCCTTTAAAGGTCGATTCTTCTCA
4	R	TTATTTTTACCGTTCCATGGTCGGAAAAATATTCATGTGCATAAAT
5	F	GTATTAAAAGTAAAATTAAATCTATATCACAATCAAATACT
6	R	AGTCTTATCAGTGAAAATGAAATCTACATTTCCTAAATCTCCAT
7	R	CGTCGTAGTCGTTCAAATTGT
8	F	ACCCAAATTCAACAGAGGTAAGT
9	R	TAGAGGATCCCCATGGAGCTTTACATAAGGAAGATTTCAACAC
10	F	AGAAGTAGAATCATCAAACCACTC
11	R	CTACCTTAAGTACGTAACTAGTACTT
12	F	TATGTATTTTTTTTGTAATTTCTGTG
13	R	GACGGCCAGTGAATTCTATTCCATGAAGAGCTAGATTTGTACTT
14	F	ATTCGATATCTATATTAATGTGTTCCAAAAGGTGC
15	R	CAATCTCGAGCAAATTCGACTGGCGATCGTTAAC
16	F	TGATTACGCCAAGCTTGCAGGCGGTAGTATTATACGTT
17	R	ATTTGGATCCAATATACTTGCTTAAGTCTTCTGAAATGTTTGTTCTTTTTTTTTG
18	F	GCAAGTATATTGGATCCAAATGTAAAAATAAAAAAAAAATAT
19	F	ACATGCACGGAGATCTAGGTAACGTTGACTTCATTTTCACTAATAAGACTGGTACCCTCA
20	R	TTGTTATTAGTGAGGGTACCAGTCTTATTAGTGAAAATGAAGTCAACGTTACCTAGATCT
21	F	ACATGCACGGAGATCTAGGTAACGTTGACTTCATTTTCACTGACAAGACTGGTACCCTCA
22	R	TTGTTATTAGTGAGGGTACCAGTCTTGTCAGTGAAAATGAAGTCAACGTTACCTAGATCT

F: Forward, R: Reverse
